# Exposure assessment of 170 pesticide ingredients and derivative metabolites in people from the Central Andes of Peru

**DOI:** 10.1038/s41598-022-17772-1

**Published:** 2022-08-08

**Authors:** Jorge Honles, Claire Clisson, Claudia Monge, Pedro Vásquez-Ocmín, Juan Pablo Cerapio, Sysay Palamy, Sandro Casavilca-Zambrano, Javier Herrera, Pascal Pineau, Eric Deharo, Vincent Peynet, Stéphane Bertani

**Affiliations:** 1 UMR 152 PHARMADEV, IRD, UPS, Université de Toulouse, Toulouse, France; 2 International Joint Laboratory of Molecular Anthropological Oncology (LOAM), IRD, INEN, Lima, Peru; 3 Institut de Recherche et d’Expertise Scientifique, Europarc, Strasbourg, France; 4 Banco Nacional de Tejidos Tumorales, Instituto Nacional de Enfermedades Neoplásicas, Lima, Peru; 5 UMR 1037 CRCT, INSERM, UPS, CNRS UMR 5071, Université de Toulouse, Toulouse, France; 6 Laboratory of Excellence Toulouse-Cancer (TOUCAN), UPS, Université de Toulouse, Toulouse, France; 7 Faculty of Pharmacy, University of Health Sciences, Vientiane, Laos; 8 UMR 260 LEDa, IRD, CNRS UMR 8007-260, Université Paris Dauphine, Paris, France; 9 Unité Organisation Nucléaire et Oncogenèse, Institut Pasteur, INSERM U 993, Paris, France; 10 UMR 224 MIVEGEC, IRD, CNRS UMR 5290, Université de Montpellier, Montpellier, France; 11 Faculté de Pharmacie, UMR 152 PHARMADEV, 35 Chemin des Maraîchers, 31000 Toulouse, France

**Keywords:** Environmental impact, Risk factors

## Abstract

The Central Andes of Peru are a region of great concern regarding pesticide risk to the health of local communities. Therefore, we conducted an observational study to assess the level of pesticide contamination among Andean people. Analytical chemistry methods were used to measure the concentrations of 170 pesticide-related compounds in hair samples from 50 adult Andean subjects living in rural and urban areas. As part of the study, a questionnaire was administered to the subjects to collect information regarding factors that increase the risk of pesticide exposure. Our results indicate that Andean people are strongly exposed to agrochemicals, being contaminated with a wide array of pesticide-related compounds at high concentration levels. Multivariate analyses and geostatistical modeling identified sociodemographic factors associated with rurality and food origin that increase pesticide exposure risk. The present study represents the first comprehensive investigation of pesticide-related compounds detected in body samples collected from people living in the Central Andes of Peru. Our findings pinpoint an alarming environmental situation that threatens human health in the region and provide a rationale for improving public policies to protect local communities.

## Introduction

The term “pesticide” refers to any chemical substance used to ward off animals or plants that are deemed harmful or undesirable. In agriculture, pesticides are used on crops at different stages to keep pest invasions at bay: herbicides are applied before seeding, fungicides during the growth stage of plants, and insecticides at the end of the growing season. Agrochemicals have made a significant contribution to feeding the world, enhancing food production and availability^1^. However, pesticides are also ubiquitous pollutants, causing adverse effects on the environment and human health^2^. A major public health concern, pesticides have been linked to disorders such as endocrine disruption and cancer^3,4^, as well as impaired neurological development in children exposed during the prenatal period and infancy^5^. People can be exposed to pesticides by dermal contact, inhalation, or ingestion of contaminated food and water^6^. Pesticide poisoning depends essentially on the duration, frequency, and concentration of exposure^7^. In this regard, agricultural workers are the most at risk of occupational exposure to pesticides due to their application and harvesting practices^6^.

Peru's agricultural sector is a major contributor to the South American food supply chain and a key component of its economy. A historical cradle of plant domestication^8,9^, the Central Andes of Peru represent an important producer of food crops and native varieties, encompassing two agroecosystems: the highlands and the coastal semiarid zones. Despite strong influences from western practices, agricultural production in this region is still dominated by smallholder farmers and traditional cropping systems, particularly in the highlands^10,11^. According to the 2012 agriculture census, 88% of Peruvian farmers use chemical pesticides regularly, while only 5% of them practice organic agriculture^12^. This figure is consistent with the higher risks of pesticide pollution reported in low- and middle-income countries (LMICs)^13^, where agrochemical regulation is not strictly enforced^14^. In this context, pesticide pollution poses a threat to the health of populations, both directly for users and those living near cultivated fields, and indirectly for people who consume contaminated food^15^. Of collective memory, the tragedy of Tauccamarca vividly exposed pesticide hazards that endanger the local communities of the Central Andes of Peru. During this event, 24 children died of acute poisoning due to the accidental mishandling of the organophosphate insecticide parathion, which caused great consternation in the population and a controversy over the toxicity of agrochemicals^16,17^. Since then, studies have reported consistent contamination of food crops cultivated in this region with pesticides, raising concerns about human health^18,19^. Meanwhile, the cancer epidemiology in the Central Andes of Peru features anomalies with divergent forms and early onsets of disease^20,21^, suggesting that anthropogenic risk factors such as pesticides might play a role in these unusual disease patterns^18,22^. Yet, the issue remains devoid of studies that directly measure the exposure of local communities to pesticide ingredients and derivative metabolites in body samples, thereby allowing a true assessment of the contamination people are subjected to.

To address this gap in knowledge, we conducted an explorative observational study to determine the level of pesticide contamination in people from the Central Andes region of Peru. We measured the concentrations of 170 pesticide ingredients and derivative metabolites in hair samples from 50 adult subjects living either in rural or urban areas. The risk of exposure to pesticides was further investigated by analyzing living space and lifestyle. For comparison, we conducted a similar study on two cohorts of people from high- and low-income countries.

## Methods

### Study design and subjects

**Figure 1:**
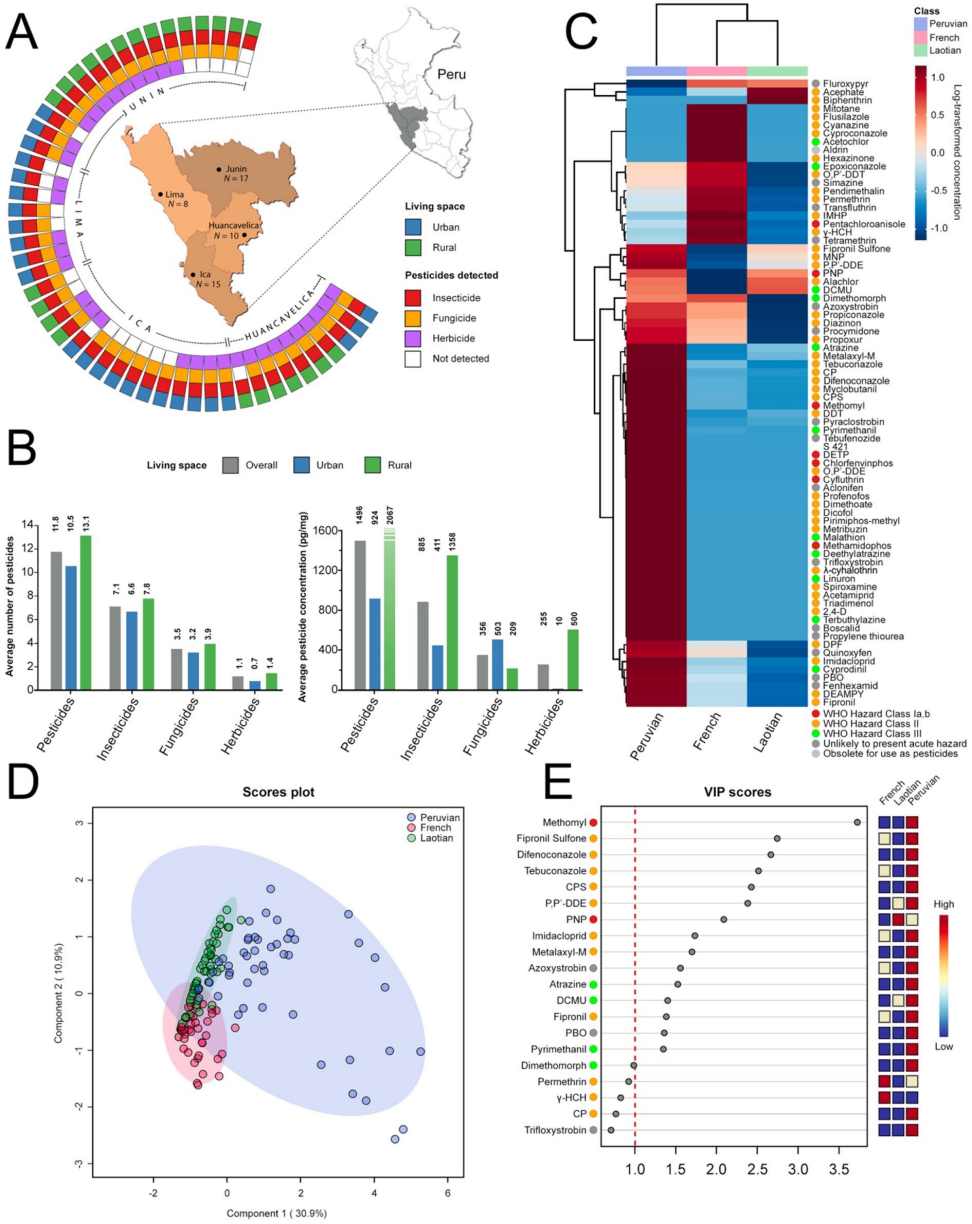
Hair samples of Peruvians from the Central Andes display higher levels of pesticide contamination. (**A**) Classification of Peruvian subjects (*N* = 50) according to the types of pesticide ingredients detected, illustrated as different concentric rings: rural or urban living space (outermost ring), followed inward by contamination with insecticides, fungicides, and herbicides (innermost ring). (**B**) Histogram showing levels of pesticide contamination in the overall cohort (grey), and in rural (green) and urban (blue) groups (both *N* = 25). (**C**) Supervised heatmap showing average concentration levels of the 76 contaminants detected (rows) across French (*N* = 47; red), Laotian (*N* = 50; green), and Peruvian (*N* = 50; blue) subjects classified in three classes (columns). Log-transformed concentrations are limned to illustrate the contamination level according to the right-hand legend. Colored dots indicate hazard classes according to the WHO recommended classification of pesticides: Class I, red; Class II, orange; Class III, green; Unlikely to present acute hazard; dark grey; Obsolete for use as pesticides, light grey. (**D**) PLS-DA score plot for the 76 contaminants detected across French (*N* = 47; light red), Laotian (*N* = 50; light green), and Peruvian (*N* = 50; light blue). Cross validation details: Accuracy = 0.61; R^2^ = 0.43; Q^2^ = 0.40. Colored areas represent 95% confidence regions. (**E**) Dot plot displaying VIP scores for the top 20 most important contaminants identified by the PLS-DA model. VIP score values: Methomyl, 3.72; Fipronil Sufone, 2.74; Difenoconazol, 2.66; Tebuconazole, 2.51; CPS, 2.42; P,P'-DDE, 2.38; PNP, 2.10; Imidacloprid, 1.73; Metalaxy-M, 1.70; Azoxystrobin, 1.56; Atrazine, 1.52; DCMU, 1.40; Fipronil, 1.38; PBO, 1.36; Pyrimethanil, 1.3; Dimethomorph, 0.98; Permethrin, 0.92; γ-HCH, 0.82; CP, 0.77; Trifloxystrobin, 0.70. The red dashed line indicates the cutoff threshold at VIP score = 1. Colored boxes on right indicate concentration association ranging from low (blue) to high (red) of the corresponding contaminant with the French, Laotian, and Peruvian classes of subjects. Colored dots on the left indicate hazard classes according to the WHO recommended classification of pesticides: Class I, red; Class II, orange; Class III, green; Unlikely to present acute hazard; dark grey.

The study was conducted between November and December 2020 among 50 adult subjects from four departments in the Central Andes of Peru: Huancavelica, Ica, Junin, and Lima (Fig. 1A and Table S1). Participants were randomly selected, respecting gender balance (1:1) and assigned into two groups according to their living space: rural (*N* = 25) and urban (*N* = 25). Along with hair sampling, a semistructured questionnaire was administered to the subjects to collect information regarding their living environment and lifestyle (Supplemental Information)^23,24^. The interview was conducted during face-to-face interviews with an epidemiologist who is a native Spanish speaker. In addition, an eTrex® 20 satellite navigation device (Garmin) was used to determine the coordinates of the survey sites. Hairs were also sampled among French (*N* = 47) and Laotians (*N* = 50) for pesticide contamination appraisals, according to the same study design and period of time (see Table S1).

### Materials

Hair collection kits were obtained from Kudzu Science (Strasbourg, France). All standards for preparing calibrators were purchased from Sigma-Aldrich (Saint-Quentin-Fallavier, France) and LGC (Molsheim, France). Acetonitrile (ACN) and methanol (MeOH) were purchased from Carlo Erba Reagents (Val-de-Reuil, France). All reagents were high-performance liquid chromatography (HPLC) grade. A blank hair matrix was obtained from a hairdresser (Strasbourg, France). Standard analyte mix stock solutions (1,000 µg mL^−1^) were prepared in ACN or MeOH, as the situation required. A deuterated internal standard stock solution consisting of 4,4′-DDT D8 (5 µg mL^−1^), Acetochlor D11 (5 µg mL^−1^), Atrazine D5 (5 µg mL^−1^), Isoproturon D6 (5 µg mL^−1^), and Simazine D10 (1 µg mL^−1^) was prepared in an ACN/MeOH mixture (50:50).

### Hair sampling and extraction process

Hair samples were collected using a hair collection kit according to the manufacturer's instructions. Briefly, a randomly selected section of hair from the head was isolated using a circled guide of 5 mm in diameter. With scissors sterilized with 70% alcohol, strips were cut as close to the scalp as possible. Hair samples were placed into an aluminum collection foil indicating the root-ends and stored dried at 4 °C for two weeks before analysis. We considered a hair growth of 1 cm/month and a length of 0.5 cm inside the scalp that cannot be sampled^25^. The three-cm long section of hair from the root-ends was cut into small sections and pounded (60 oscillations/min) in a mixer mill MM400 (Retsch). Fifty milligrams of hair powder were suspended in 1000 μL of organic solvent consisting of a mixture of ACN/MeOH (50:50), and 50 μL of internal standard stock solution were added. The suspensions were sonicated at room temperature for 15 min with a VWR® ultrasonic cleaner and then centrifuged for 5 min at 4000×*g* with a Megafuge™ 16 centrifuge (Thermo Fisher Scientific). Supernatants were collected for pesticide detection and quantification by liquid chromatography with tandem mass spectrometry (LC–MS/MS) and gas chromatography with tandem mass spectrometry (GC–MS/MS). Information on hair samples is detailed in Table S1 (sampling date, mass, length, hair coloring, and estimated exposure period).

### Analytical methods

For LC–MS/MS, samples were acquired on a 1290 Infinity liquid chromatography (LC) system coupled to a 6460 triple quadrupole mass spectrometer (MS) with a G4226A 1290 autosampler (all Agilent Technologies). Chromatographic separations were performed on a Nucleodur C18 HTec column (100 × 2 mm I.D.; 1.8 µm) (Macherey–Nagel). The mobile phase gradient consisted of water containing 0.1% formic acid (solvent A) and ACN (solvent B). A flow rate of 0.7 mL/min was applied using the following conditions: (i) isocratic elution with 95% A and 5% B from the injection time until 1 min, (ii) a gradient of 5% to 73% B over the next 11 min, and finally (iii) a further linear gradient of 73% to 90% B over 30 s. The condition returned to its initial state after 1.5 min, followed by a re-equilibration for another 1.5 min. The column temperature was set at 50 °C, the autosampler temperature at 5 °C, and the injection volume was 5 μL. MS was interchangeably equipped with either a G1958-65138 Jet Stream electrospray ionization (ESI) source or with an atmospheric pressure chemical ionization (APCI) source (both Agilent Technologies) operating in multiple reaction monitoring (MRM) mode. Mass detection was performed in positive ionization (PI) and negative ionization (NI) modes at nominal resolution. The nebulization gas was heated to 300 °C with a flow rate of 5 L/min, and the nebulizer pressure was 45 psi. The sheath gas was heated to 300 °C with a flow rate of 10 L/min, and the ionization spray voltage was 3.5 kV (for both PI and NI). Two or three main transitions for each standard were performed in PI and NI modes. A curve of calibration was obtained with a mix of standards at final concentrations ranging from 0.2 ng/mL to 200 ng/mL in ACN/MeOH (50:50).

For GC–MS/MS, samples were acquired on a 7890A gas chromatograph (GC) system with a multimode inlet (MMI) coupled to a 7000B triple quadrupole MS (both Agilent Technologies) with multipurpose autosampler (Gerstel) and an HP-5 ms Ultra Inert column (30 m × 0.25 mm I.D.; 0.25 µm) (Agilent Technologies). The linear gradient consisted of an initial temperature at 60 °C for 2 min, followed with a 60 °C/min ramp rate up to 180 °C, then a 5 °C/min ramp rate up to 240 °C, then a 60 °C/min ramp rate up to 300 °C, and finally 300 °C for 4 min. The injector was equipped with a Gooseneck Splitless liner (4 mm I.D.) (Restek) and heated to 300 °C. The injection volume was fixed at 4 μL in the splitless mode, the carrier gas (helium) had a flow rate of 1 mL/min, and the transfer line was set at 300 °C. MS was equipped with a G7000-65710 inert electron impact (EI) source (Agilent Technologies) operating in MRM mode. The collision gases were helium and nitrogen (purity ≥ 99.9999%). The source was heated to 230 °C. Two or three main transitions for each standard were performed in PI and NI modes. A curve of calibration was obtained with a mix of standards at final concentrations ranging from 0.2 to 200 ng/mL in ACN/MeOH (50:50).

### Pesticide detection and quantification

The calibration curves spanned the range of biologically relevant concentrations expected in hair using a blank hair matrix that has been checked for low pesticide contamination (10 measurement points from 4 to 4000 pg/mg of blank hair matrix). Randomized samples spiked with deuterated internal standards were acquired on LC–MS/MS and GC–MS/MS systems with calibration checks performed every ten samples during the batch run. Analytes were identified based on their retention time and the ratio between the qualifier and quantifier signals. Quantification was assessed based on calibration curves obtained from adjacent calibration checks.

A total of 170 pesticide ingredients and derivative metabolites were tested in the hair matrices, including 32 fungicide ingredients and four derivative metabolites, 34 herbicide ingredients and five derivative metabolites, 66 insecticide ingredients and 26 derivative metabolites, as well as two synergistic agents and one multi-use pesticide (Table S2). A sample was found to be contaminated when the concentration of the contaminant was above the limit of detection (LOD) of the analytical method but below the limit of quantification (LOQ), i.e., the concentration of the lowest calibrator. LOD and LOQ were measured based on signal to noise ratio at about 3 and 10, respectively. The average concentration of a contaminant was calculated considering a value of zero in the samples < LOD and a value of LOQ/$\sqrt{2}$ in samples < LOQ^26^. Detailed information on analytes tested is provided in Table S2 (CAS number, quantification method, and limits of measurement).

### Statistics

Data were inputted into Excel software version 16.16.27 (Microsoft) before being transferred to PostgreSQL relational database management system version 13.3 (PostgreSQL Global Development Group). Statistical analysis was computed using the R software environment version 4.0.5 (R Core Team) and Stata software for statistics and data science version 14.0 (StataCorp). Statistical tests were performed with a 0.05 significance level. Missing data was addressed by the method of multiple imputation using the R package mice version 3.14.0^27^. Principal component analysis (PCA), partial least squares-discriminant analysis (PLS-DA), and orthogonal partial least squares-discriminant analysis (OPLS-DA) were performed using the R exploratory multivariate data analysis ropls package version 1.26.4^28^. Geospatial data were processed with QGIS geographic information system version 3.18.2-3 (QGIS Development Team) and the leafletR package for R version 0.4-0. Bayesian spatial modeling of geostatistical data was performed using the integrated nested Laplace approximation (INLA) in the R-INLA package version 21.06.11^29^. Figures were charted using Prism software version 9 (GraphPad). Geocoded information on agrochemical use in Peru was obtained from the 2019 National Agricultural Survey data openly available at: https://www.inei.gob.pe/media/DATOS_ABIERTOS/ENA/DATA/2019.zip.

### Ethics approval

The study was conducted in strict accordance with the ethical principles contained in both the Declaration of Helsinki and the Singapore Statement on research integrity. People were duly informed about the purpose and conduct of the study. Participation was voluntary with no compensation. Participants provided written informed consent for their information and data to be stored and used for research. The Institutional Review Board of INEN approved the study (Project N° 113-2014-CIE-INEN). In addition, human research ethics committees in France (CPP Sud-Est III) and in Laos (NECHR) also approved the conduct of the study (Projects N° 2020-043B and 2020.49.MC, respectively).

## Results

### Survey Data

Table 1 shows an overview of the socio-demographic characteristics of the 50 adults originating from the Central Andes of Peru, 50% of whom live in urban areas and the other 50% in rural zones (Fig. 1A). The mean ages of the subjects were 38.7 ± 15.6 years old in the urban group and 50 ± 13.4 years old in the rural group (*p* < 0.05). Both groups were matched for gender (*p* > 0.05). Farmer (36%) and merchant (22%) occupations were the most common, followed by students (8%), vehicle drivers, security officers, and technicians (all 6%), handypersons and homemakers (both 4%), and finally, mechanic, teacher, toxicologist, and waitress (all 2%). Farmers made up the vast majority (72%) of the rural group, as expected (*p* < 0.05 vs. urban group). A majority of individuals (58%) reported living within proximity to agriculture (including 8% who combined farming and mining), a trend that was more pronounced among the rural group (92%) (*p* < 0.05 vs. urban group). Pesticides were used by 54% of subjects regularly, with 34% for occupational purposes (all in the rural group) and 18% for domestic purposes (*p* < 0.05; rural group vs. urban group). Besides, 35% of professional users said they did not wear personal protective equipment (PPE) when applying pesticides, and no one handling pesticides occasionally at home did so. Participants claimed to get their feed from the market (66%), but the rural group was much more likely to consume crops directly harvested from fields (68%) and natural water sources (80%) (both *p* < 0.05 vs. urban group). Of note, 10% of the subjects reported dyeing or bleaching their hair within the three months before the study (Table S1). Furthermore, 82% of the subjects stated they were not taking any medication at the time of sampling or during the period before (*p* > 0.05; rural group vs. urban group), thereby ruling out the possibility of pharmaceutical metabolites meddling with the analyses for pesticide search.

**Table 1:** Baseline socio-demographic characteristics of the 50 adult Peruvian participants in the study.

Feature	Overall	Urban	Rural	*p* value (urban vs. rural)
**Cohort**	50 (100%)	25 (100%)	25 (100%)	
**Age (years)**				0.005*
Mean ± SD	44.4 ± 15.5	38.7 ± 15.6	50 ± 13.4	
Median	44.5	41	50	
Range	[19–69]	[19–68]	[26–69]	
Interquartile range	28.7	26	23.5	
**Gender**				1**
Female	25 (50%)	12 (48%)	13 (52%)	
Male	25 (50%)	13 (52%)	12 (48%)	
**Department of origin**				6.3E−5***
Huancavelica	10 (20%)	3 (12%)	7 (28%)	
Ica	15 (30%)	11 (44%)	4 (16%)	
Junin	17 (34%)	3 (12%)	14 (56%)	
Lima	8 (16%)	8 (32%)	0 (0%)	
**Occupation**				3.8E−8***
Farmer	18 (36%)	0 (0%)	18 (72%)	
Merchant	11 (22%)	9 (36%)	2 (8%)	
Other	21 (42%)	16 (64%)	5 (20%)	
**Medication intake**				0.74***
Yes	4 (8%)	1 (4%)	3 (12%)	
No	41 (82%)	21 (84%)	20 (80%)	
Undetermined	5 (10%)	3 (12%)	2 (8%)	
**Activity in the immediate environment**				1.40E−6***
Agribusiness	1 (2%)	1 (4%)	0 (0%)	
Farming	25 (50%)	6 (24%)	19 (76%)	
Farming and mining	4 (8%)	0 (0%)	4 (16%)	
None	20 (40%)	18 (72%)	2 (8%)	
**Origin of the food consumed**				2.2E−7***
Farm (harvested)	17 (34%)	0 (0%)	17 (68%)	
Market (bought)	33 (66%)	25 (100%)	8 (32%)	
**Source of water consumed**				7.5E−5**
Artificial (bottled, tapped, etc.)	25 (50%)	20 (80%)	5 (20%)	
Natural (river, rain, well, etc.)	25 (50%)	5 (20%)	20 (80%)	
**Occupational use of pesticides**				2.2E−7***
Yes	17 (34%)	0 (0%)	17 (68%)	
No	33 (66%)	25 (100%)	8 (32%)	
**Domestic use of pesticides**				0.002***
Yes	9 (18%)	9 (36%)	0 (0%)	
No	41 (82%)	16 (64%)	25 (100%)	
**Use of PPE (only pesticide users)**				0.004***
Yes	9 (35%)	0 (0%)	9 (53%)	
No	15 (57%)	9 (100%)	6 (35%)	
Undetermined	2 (8%)	0 (0%)	2 (12%)	

Mean values are presented ± standard deviation (SD). Percentages are expressed as ratios of the individuals considered in the parameter with: Pesticide users, *N* = 26; Rural pesticide users, *N* = 17; Urban Pesticide users, *N* = 9.

*t-test; **χ^2^ test; ***Fisher's exact test.

### Pesticide contamination in Andean hair samples and comparative evaluation

Of the 170 compounds tested, a total of 67 were detected (> LOD) and 51 were quantified (> LOQ) in the hair samples of the 50 adult Andean subjects (Table S3). Table 2 summarizes the results for the 67 contaminants detected in Peruvian samples, including 24 insecticide ingredients and nine derivative metabolites, 17 fungicide ingredients and one derivative metabolite), 10 herbicide ingredients and one derivative metabolite, two multi-use ingredients and one derivative metabolite, and two synergist agents. Overall, the average number of contaminants detected per sample was 11.8 ± 6.2 (ranging between 3 and 27), with a total average concentration of 1496 ± 2796 pg/mg of hair (Fig. 1B).

**Table 2:** Descriptive statistics for the pesticide contaminants found in the hair of the 50 adult Peruvian participants in the study.

Pesticide			Occurrence (*N* = 50)			Statistical distribution (pg/mg)					
						Percentile score					Maximum concentration
Name	Type	Hazardous class	D	Q	Q + D	0.10	0.25	0.50	0.75	0.90	
2-(Diethylamino)-6-methyl-1H-pyrimidin-4-one (DEAMPY)	Insecticide	II	3	0	3	ND	ND	ND	ND	ND	< LOQ
2-Isopropyl-6-methyl-4-pyrimidinol (IMHP)	Insecticide	II	0	2	2	ND	ND	ND	ND	ND	57.3
2,4-Dichlorophenoxyacetic acid (2,4 D)	Herbicide	II	0	1	1	ND	ND	ND	ND	ND	2918.6
3-Methyl-4-nitrophenol (MNP)	Insecticide	II	6	2	8	ND	ND	ND	ND	< LOQ	48.6
4-Nitrophenol (PNP)*	Insecticide	Ia	15	17	32	ND	ND	< LOQ	24.0	52.4	888.5
Acephate	Insecticide	II	0	1	1	ND	ND	ND	ND	ND	666.6
Acetamiprid	Insecticide	II	1	4	5	ND	ND	ND	ND	ND	11.6
Aclonifen	Herbicide	U	2	0	2	ND	ND	ND	ND	ND	< LOQ
Alachlor	Herbicide	II	2	0	2	ND	ND	ND	ND	ND	< LOQ
Atrazine	Herbicide	III	4	11	15	ND	ND	ND	< LOQ	5.4	1081.9
Azoxystrobin	Fungicide	U	7	15	22	ND	ND	ND	4.9	85.2	4720.2
Boscalid	Fungicide	U	0	3	3	ND	ND	ND	ND	ND	1051.9
Chlorfenvinphos	Insecticide	Ib	1	0	1	ND	ND	ND	ND	ND	< LOQ
Chlorpyrifos (CPS)	Insecticide	II	8	17	25	ND	ND	ND	29.3	160	4941.8
Clofenotane (DDT)*	Insecticide	II	0	2	2	ND	ND	ND	ND	ND	103.3
Cyfluthrin	Insecticide	Ib	1	0	1	ND	ND	ND	ND	ND	< LOQ
Cypermethrin (CP)	Insecticide	II	11	3	14	ND	ND	ND	< LOQ	< LOQ	275.2
Cyprodinil	Fungicide	III	3	3	6	ND	ND	ND	ND	< LOQ	34.2
Deethylatrazine	Herbicide	III	3	1	4	ND	ND	ND	ND	ND	8.8
Diazinon	Insecticide	II	3	2	5	ND	ND	ND	ND	ND	37.6
Dicofol	Insecticide	II	0	1	1	ND	ND	ND	ND	ND	25.5
Diethyl hydrogen phosphate (DPF)	Insecticide	II	2	2	4	ND	ND	ND	ND	ND	44.6
Difenoconazole	Fungicide	II	11	14	25	ND	ND	ND	5.5	121.5	1064.0
Dimethoate	Insecticide	II	4	0	4	ND	ND	ND	ND	ND	< LOQ
Dimethomorph	Fungicide	III	2	7	9	ND	ND	ND	ND	31.2	244.9
Diuron (DCMU)	Herbicide	III	1	15	16	ND	ND	ND	5.4	11.6	63.3
Epoxiconazole	Fungicide	III	0	5	5	ND	ND	ND	ND	ND	35
Fenhexamid	Fungicide	U	6	1	6	ND	ND	ND	ND	< LOQ	111.8
Fipronil	Insecticide	II	8	22	30	ND	ND	< LOQ	15.7	53.6	861
Fipronil Sulfone	Insecticide	II	15	35	50	< LOQ	< LOQ	6.0	11.7	20.1	104.3
Imidacloprid	Insecticide	II	12	9	21	ND	ND	ND	< LOQ	72.7	3426.5
Lambda-cyhalothrin (λ-cyhalothrin)	Insecticide	II	7	1	8	ND	ND	ND	ND	< LOQ	43.7
Lindane (γ-HCH)*	Insecticide	II	3	1	4	ND	ND	ND	ND	ND	41.5
Linuron	Herbicide	III	0	1	1	ND	ND	ND	ND	ND	5380.6
Malathion	Insecticide	III	2	3	5	ND	ND	ND	ND	ND	171.6
Metalaxyl-M	Fungicide	II	8	10	18	ND	ND	ND	< LOQ	22.4	338.2
Methamidophos*	Insecticide	Ib	2	4	6	ND	ND	ND	ND	< LOQ	1636.6
Methomyl	Insecticide	Ib	5	30	35	ND	ND	4.7	10.8	30.0	2572.8
Metribuzin	Herbicide	II	2	2	4	ND	ND	ND	ND	ND	319.7
Myclobutanil	Fungicide	II	1	1	2	ND	ND	ND	ND	ND	10.4
O,O-Diethyl hydrogen thiophophate (DETP)	Insecticide	Ia	1	0	1	ND	ND	ND	ND	ND	< LOQ
O,P'-DDE*	Insecticide	II	1	0	1	ND	ND	ND	ND	ND	< LOQ
O,P'-DDT*	Insecticide	II	2	0	2	ND	ND	ND	ND	ND	< LOQ
Octachlorodipropyl ether (S 421)	Synergist	Unknown	1	0	1	ND	ND	ND	ND	ND	< LOQ
P,P'-DDE*	Insecticide	II	15	23	38	ND	< LOQ	< LOQ	13.3	26.7	87.8
Pendimethalin	Herbicide	II	2	0	2	ND	ND	ND	ND	ND	< LOQ
Pentachloroanisole*	Fungicide	Ib	1	0	1	ND	ND	ND	ND	ND	< LOQ
Permethrin	Insecticide	II	6	0	6	ND	ND	ND	ND	< LOQ	< LOQ
Piperonyl butoxide (PBO)	Synergist	U	3	13	16	ND	ND	ND	109.1	421.5	4246.3
Pirimiphos-methyl	Insecticide	II	1	2	3	ND	ND	ND	ND	ND	6.8
Procymidone	Multi-use	U	1	4	5	ND	ND	ND	ND	ND	124.7
Profenofos	Insecticide	II	2	0	2	ND	ND	ND	ND	ND	< LOQ
Propiconazole	Fungicide	II	1	4	5	ND	ND	ND	ND	ND	43.4
Propoxur	Insecticide	II	9	3	12	ND	ND	ND	ND	< LOQ	7.8
Propylene thiourea	Fungicide	U	1	1	2	ND	ND	ND	ND	ND	105.2
Pyraclostrobin	Fungicide	U	5	2	7	ND	ND	ND	ND	< LOQ	6.7
Pyrimethanil	Fungicide	III	3	11	14	ND	ND	ND	< LOQ	16.7	93.8
Quinoxyfen	Fungicide	U	2	0	2	ND	ND	ND	ND	ND	< LOQ
Simazine	Herbicide	U	1	2	3	ND	ND	ND	ND	ND	22.6
Spiroxamine	Fungicide	II	1	3	4	ND	ND	ND	ND	ND	33.2
Tebuconazole	Fungicide	II	8	19	27	ND	ND	< LOQ	10.1	78.1	172.9
Tebufenozide	Insecticide	U	1	0	1	ND	ND	ND	ND	ND	< LOQ
Terbuthylazine	Herbicide	III	0	1	1	ND	ND	ND	ND	ND	4.2
Tetramethrin	Insecticide	U	2	7	9	ND	ND	ND	ND	94.6	353.1
Transfluthrin	Insecticide	U	2	1	3	ND	ND	ND	ND	ND	51.8
Triadimenol	Fungicide	II	1	1	2	ND	ND	ND	ND	ND	43.5
Trifloxystrobin	Fungicide	U	5	4	9	ND	ND	ND	ND	< LOQ	22.8

D, number of subjects for whom analyte was only detected; Q, number of subjects for whom analyte was quantifiable; Q + D, number of subjects for whom analyte was either quantified or detected; ND, not detected. Hazardous classes are defined according to the WHO recommended classification of pesticides by hazard (2019 edition), such as: Class Ia, extremely hazardous; Class Ib, highly hazardous; Class II, moderately hazardous; Class III, slightly hazardous; U, unlikely to present acute hazard.

*Pesticide ingredients and derivative metabolites banned in Peru.

For comparison, the average numbers of contaminants detected in hair samples from French and Laotian subjects were 4.3 ± 3.5 and 3.2 ± 1.6 (both ranging between 0 and 16), respectively (*p* = 1.26E−10 and 2.49E−13 vs. Peruvian samples) (Fig. 1C). Twenty-five out of the 76 contaminants detected across all three classes of subjects were only found in Peruvian hair samples (32.9%). Likewise, the average concentration of pesticides in French samples was 113.9 ± 138.8 pg/mg of hair, while it was 16.8 ± 43.1 pg/mg of hair in Laotian samples (*p* = 0.001 and 0.0005 vs. Peruvian samples, respectively). To illustrate this difference in pesticide exposure, the lower quartile of Peruvian subjects had higher levels of contaminants in their hair samples than those in the upper quartile of both French and Laotian subjects (Fig. S1). Moreover, variable influence on projection (VIP) obtained using a PLS-DA model identified that methomyl, fipronil and its derivative fipronil sulfone, difenoconazole, tebuconazole, chlorpyrifos (CPS), P,P'-DDE, imidacloprid, metalaxyl-M, azoxystrobin, atrazine, diuron (DCMU), piperonyl butoxide (PBO), and pyrimethanil were significantly associated with Peruvians, as opposed to French and Laotians (VIP score > 1) (Fig. 1D, E and Table S4 for PLS-DA model performance).

### Pesticide Identification in Andean hair samples

**Figure 2:**
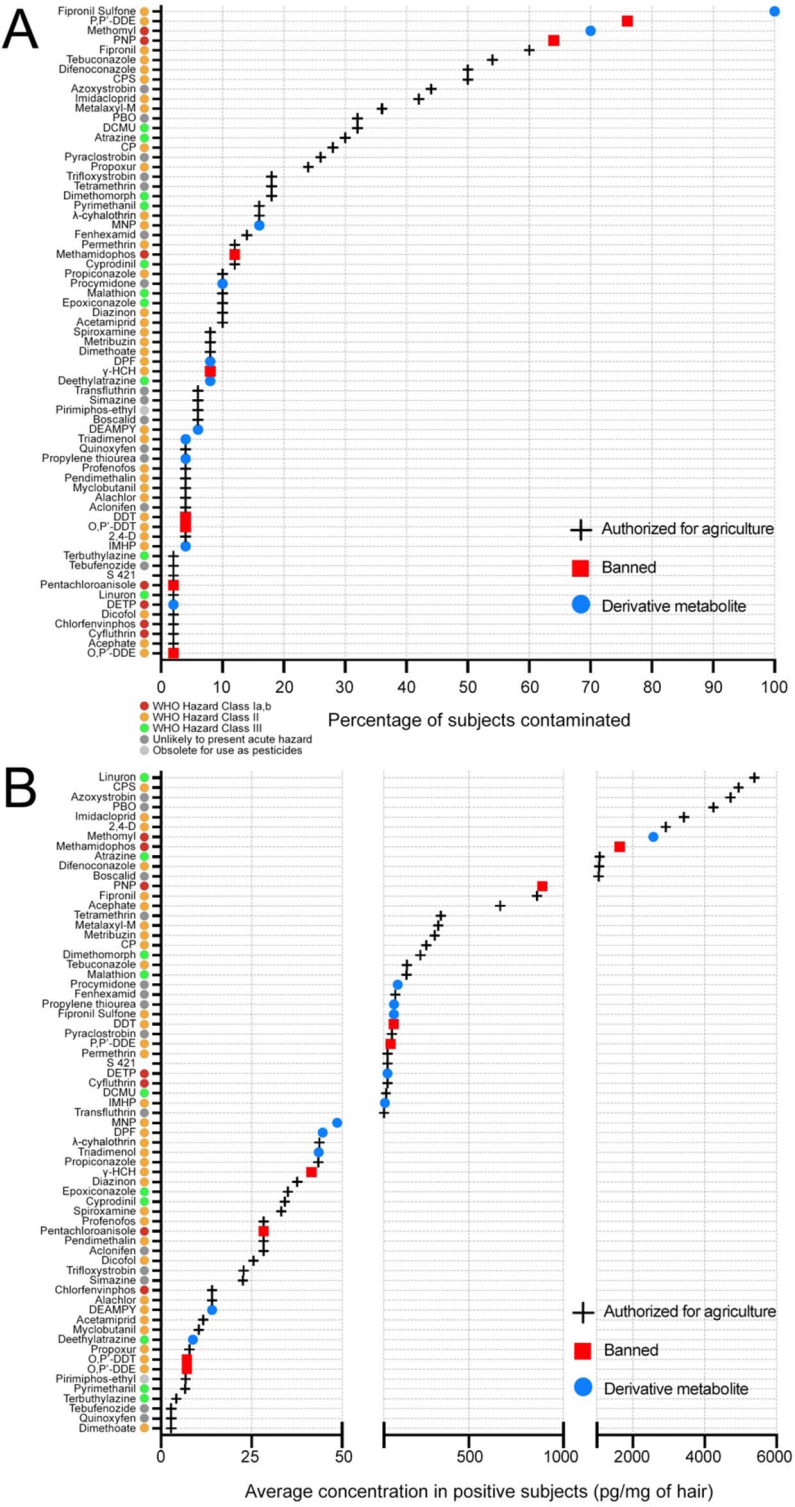
Hair samples of Peruvians from the Central Andes are contaminated with a wide range of pesticides. (**A**) Dot plot displaying the percentage of subjects (*N* = 50) exposed to each of the 67 contaminants detected. (**B**) Dot plot displaying the average concentration (pg/mg of hair) in positive subjects (> LOQ) for each of the 67 contaminants. (**A**,**B**) Crosses: pesticides authorized for agriculture use in Peru; Red squares: pesticide ingredients legally banned in Peru; Blue dots: Pesticide derivative metabolites. Colored dots indicate hazard classes according to the WHO recommended classification of pesticides: Class I, red; Class II, orange; Class III, green; Unlikely to present acute hazard; dark grey; Obsolete for use as pesticides, light grey.

**Figure 3:**
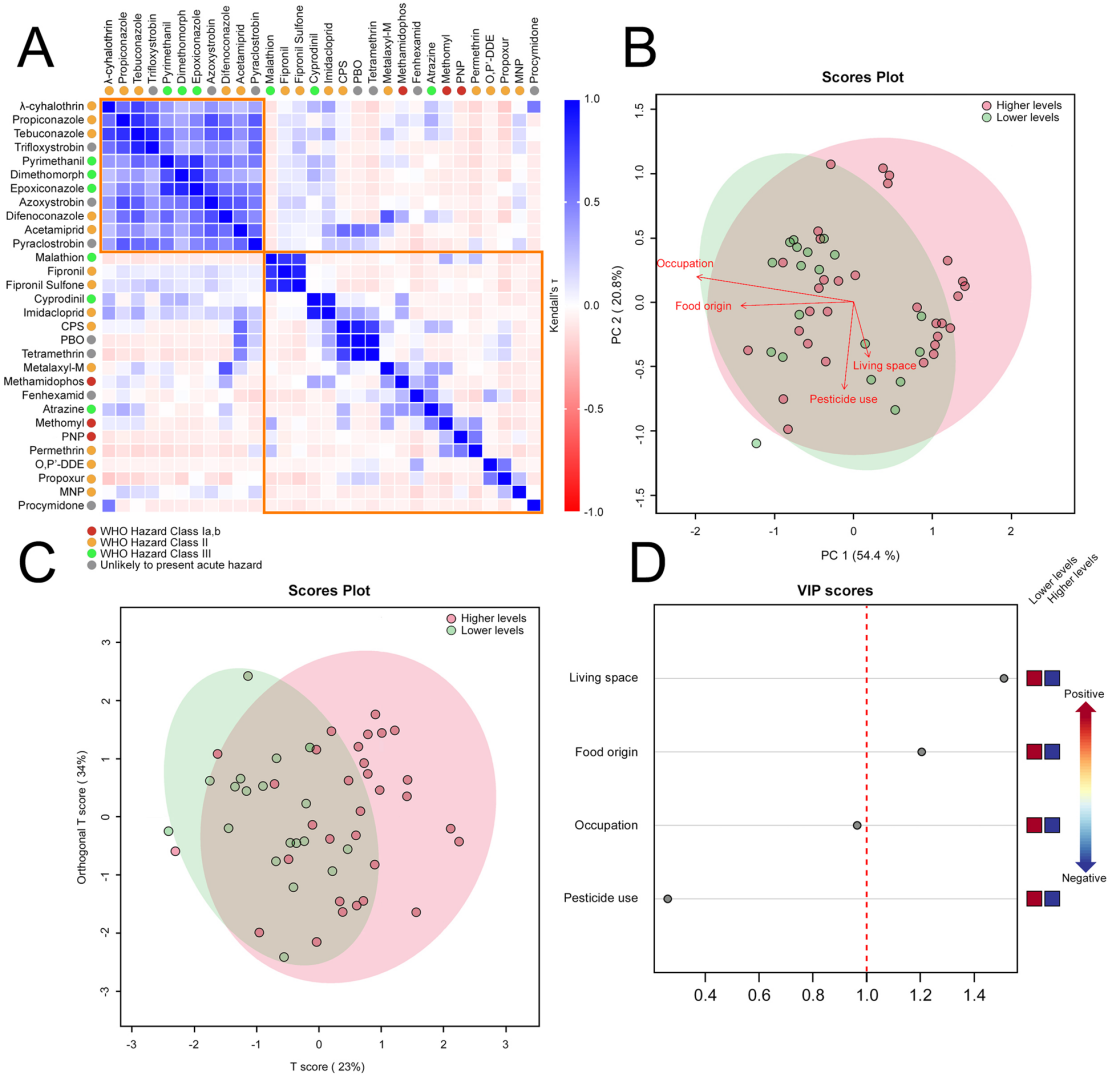
High levels of pesticide contamination in hair samples in Peruvians from the Central Andes are associated with lifestyle traits. (**A**) Sorted correlation matrix of the 30 contaminants with the highest average concentrations in hair samples (pg/mg). Kendall's τ coefficients are limned to illustrate the significance of the correlation according to the right-hand legend. Colored dots indicate hazard classes according to the WHO recommended classification of pesticides: Class I, red; Class II, orange; Class III, green; Unlikely to present acute hazard; dark grey. (**B**,**C**). Multivariate score plots for relevant sociodemographic variables in high- (red) and low- (green) contaminated subjects (*N* = 50) [with high contamination defined herein as number of pesticides > 10.5 (median) or average pesticide concentration > 346 pg/mg of hair (median)]. (**B**) PCA score plot. Colored areas represent 95% confidence regions. (**C**) OPLS-DA score plot (R^2^X = 0.23; R^2^Y = 0.24; Q^2^ = 0.12). Colored areas represent 95% confidence regions. (**D**) Dot plot displaying VIP scores for each sociodemographic variable included in the OPLS-DA model. VIP score values: Living space, 1.51; Food origin, 1.20; Occupation, 0.96; Pesticide use, 0.26. The red dashed line indicates the cutoff threshold at VIP score = 1. Colored boxes on right indicate direction of association of the corresponding sociodemographic factor with high- and low-contaminated subjects.

More than 60% of the Peruvian hair samples revealed contamination by clofenotane (DDT) and its derivative metabolites (i.e., O,P'-DDE, O,P'-DDT, and P,P'-DDE), fipronil and its derivative fipronil sulfone, parathion's metabolite 4-nitrophenol (PNP), and thiodicarb's metabolite methomyl (Fig. 2A and Table 2). Of note, fipronil sulfone was detected in 100% of samples, with an average concentration of 10.5 ± 15.5 pg/mg of hair. Secondarily, azoxystrobin, CPS, difenoconazole, imidacloprid, and tebuconazole were detected in over 40% of samples. Additionally, atrazine, cypermethrin (CP), DCMU, metalaxyl-M, PBO, propoxur, and pyrimethanil were spotted in more than 20% of samples; the remaining contaminants in less than 20%. In terms of quantification, PNP, azoxystrobin, CPS, difenoconazole, fipronil, methomyl, PBO, tebuconazole, and tetramethrin were the contaminants with the highest concentrations in positive subjects (i.e., average concentration > percentile score 0.75) (Fig. 2B and Table 2). Noticeably, several pesticides or derivative metabolites whose use is prohibited in Peru were found (Fig. 2A,B and Table 2). This is notably the case for PNP, DDT, pentachloroanisole, and lindane (γ-HCH), as well as methamidophos which was banned only a few months before the conduct of our study (see Table S2). For example, PNP and methomyl were detected in more than 60% of the Peruvian subjects and quantified in positive individuals at high average concentrations with 67.2 ± 162.2 and 214.6 ± 602 pg/mg of hair, respectively (Table S3). The correlation matrix including the 30 analytes with the highest average concentrations revealed a certain degree of coherence regarding multi-contamination and pesticide application practices: insecticide derivative metabolites clustered with their parent pesticide ingredients; whereas fungicide compounds were grouped according to their chemotype (Fig. 3A).

### Effect of socio-demographic factors on pesticide contamination in Andean people

Table 3 presents the bivariate descriptive statistics relating pesticide contamination to socio-demographic factors (presented in Table 1). Living space (rural vs. urban), occupation (farmer vs. other), and the origin of the food consumed (farm vs. market) were the variables significantly associated with the number of pesticides detected in hair samples; whereas pesticide use barely reached the level of significance (*p* < 0.05). Similarly, occupation, the origin of the food consumed, and pesticide use were significantly correlated with the concentration of contaminating pesticides, but at lower statistical degrees (*p* < 0.05). Following the bivariate analysis, the four variables retained were introduced into a supervised multivariate analysis to reveal a more comprehensive picture of the impact of each factor on pesticide contamination level. PCA and OPLS-DA models indicated that the socio-demographic factors associated with the highest levels of pesticide contamination (VIP score > 1) were living in rural areas and eating food harvested directly from the field (VIP scores = 1.51 and 1.20, respectively); albeit the cumulative value (cum) of R^2^X, R^2^Y and Q^2^ indicates a limited fit and prediction ability of the OPLS-DA model (cum < 1) (Fig. 3B–D and Table S4 for PCA and OPLS-DA models performance).

**Table 3:** Results of the bivariate analysis between socio-demographic factors and pesticide contamination in the hair of the 50 adult Peruvian participants in the study.

Feature	Number of pesticides					Pesticide concentration			
	*N*	Mean	SD	t-score	*p* value	Mean (pg/mg)	SD	t-score	*p* value
**Age (years)**				0.2	0.401			1.2	0.122
[19–44]	25	12	7.1			101.9	139.5		
[45–69]	25	11.6	5.2			73.5	108		
**Gender**				1.3	0.105			1.5	0.069
Female	25	11	6.4			59.8	80.4		
Male	25	12.6	5.9			115.6	153.2		
**Living space**				1.9	0.034			1	0.162
Rural	25	13.1	5.7			112.9	155.3		
Urban	25	10.5	6.4			62.5	78.1		
**Occupation**				2.6	0.007			1.8	0.035
Farmer	18	14.3	5.8			143.7	173.7		
Other	32	10.3	6			56.2	71.1		
**Activity in the immediate environment**				0.97	0.169			1.18	0.121
Farming	29	12.3	5.8			110	148.2		
Other	21	11.1	6.7			57	73.4		
**Origin of the food consumed**				3.2	0.001			1.8	0.035
Farm	17	15	5.2			148.6	177.6		
Market	33	10.1	6			56.3	70.1		
**Source of water consumed**				0.3	0.398			1.2	0.115
Artificial	25	12.1	6.5			71.5	116.9		
Natural	25	11.5	5.9			103.9	131.7		
Use of pesticides*				1.8	0.041			17	0.049
Yes	26	12.9	5.6			119	153.7		
No	24	10.5	6.6			53.8	70.2		
**Use of PPE (only pesticide users)**				1.2	0.125			− 1.9	0.963
Yes	9	14.1	5.5			200.8	188.5		
No	15	11.4	5.1			58.6	71.3		

*t*-tests were performed with log-transformed mean values.

*Defined herein as either occupational or domestic use of pesticides.

### Geospatial distribution of pesticide contamination in the Central Andes of Peru

**Figure 4:**
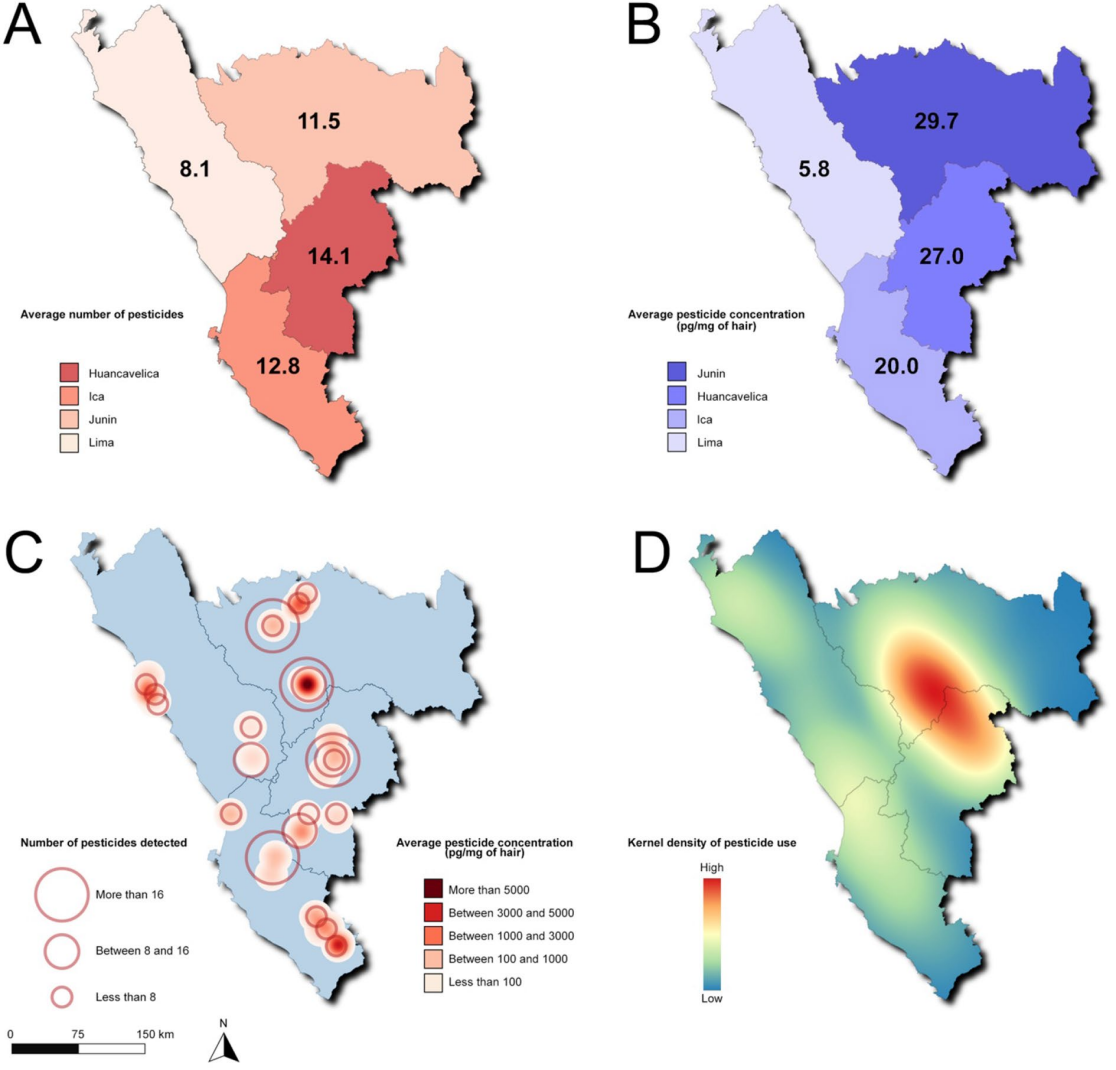
Spatial distribution of pesticide contamination among Peruvians from the Central Andes. (**A**) Choropleth map showing the average number of pesticides per subject according to their home department (red shades). (**B**) Choropleth map showing the average pesticide concentration (pg/mg of hair) per subject according to their home department (blue shades). (**C**) Proportional symbol map showing the spatial distribution of the subjects (*N* = 50). Circle area and color intensity (red shades) denote the number and the concentration of contaminating pesticides in hair samples, respectively. (**D**) Kernel density map showing pesticide use in the Central Andes of Peru, according to the 2019 National Agricultural Survey. Colors indicate the gradient in pesticide use intensity ranging from blue (low) to red (high).

Subjects were georeferenced and spatially analyzed according to their living space and pesticide contamination. Choropleth mapping revealed that the highest levels of pesticide contamination in hair samples were found in Huancavelica and Junin, followed by Ica and then Lima (Fig. 4A,B). This figure was confirmed at a finer granularity when mapping individuals: the Mantaro valley between Junin and Huancavelica encompassed the subjects who were exposed to the widest array of pesticides at the highest concentration levels (Fig. 4C). Interestingly, the spatial distribution in pesticide contamination overlapped with the density of agrochemical use in the Central Andes of Peru (Fig. 4D). Such observation was further confirmed by the fact that a Bayesian spatial model with random effects, in which we incorporated geocoded data on pesticide contamination and agrochemical use, demonstrated geostatistical significance with an intercept precision mean of 63.06 (confidence interval: 63.04–63.08). Performance statistics of the INLA model is provided in Table S4.

## Discussion

In the present study, we conducted an explorative observational investigation to assess pesticide exposure among people from the Central Andes of Peru. Despite several studies conducted in the region reporting pesticide contamination of food crops and persistent organic pollutants (POPs) in the environment^18,30^, our study represents, to the best of our knowledge, the first comprehensive analysis of pesticide-related contamination in body samples from Andean people. We measured the concentrations of 170 active ingredients and derivative metabolites in hair samples from 50 adult Andean individuals living in rural and urban areas, using validated analytical methods^31^. Hair matrix has proven to be efficient in assessing pesticide exposure over a period covering weeks to months^32,33^, despite not providing toxicokinetic information. Moreover, the hair matrix offers another advantage in epidemiological studies, as it is not sensitive to intraday variabilities like urine and plasma compartments^34,35^. However, there is hitherto no reference range available to classify pesticide poisoning in the hair so that we can congruently estimate the level of intoxication Andean people face. Therefore, we compared pesticide contamination levels found in Peruvian hair samples with those measured in French and Laotian individuals, taken herein as avatars for high- and low-income countries, respectively.

Our results indicate that people living in the Central Andes of Peru are particularly at risk from pesticides. Analytical chemistry revealed that Andean people are consistently exposed to a wide array of 67 pesticide-related products (25 of them exclusively found in Peruvian samples), with the highest levels of pesticide contamination in hair samples exceeding 10,000 pg/mg. Overall, hair samples from Andean subjects contained an average of 11.8 pesticide-related products at a mean concentration of 1,496 pg/mg. These numbers are significantly higher compared to the amounts of pesticides recorded in French and Laotian subjects. About 50.7% of the contaminants detected in Peruvians pertained to insecticide ingredients, 28.3% to fungicide ingredients, and 16.4% to herbicide ingredients, while the remainder were synergists or multi-purpose agents. According to WHO^36^, two compounds detected in Andean hair samples are extremely hazardous (Class Ia), five are highly hazardous (Class Ib), 35 are moderately hazardous (Class II), 10 are slightly hazardous (Class III), and 14 are unlikely to present an acute hazard; one compound remains undetermined.

Based on our analysis, we further identified two groups of contaminants that are of particular concern regarding pesticides' impact on local human health. The first group of contaminants, found at significantly higher levels in Peruvian subjects than in French and Laotian subjects, includes azoxystrobin, CPS, difenoconazole, fipronil and its derivative fipronil sulfone, methomyl, PBO, and tebuconazole. The second group of contaminants consists of PNP, tetramethrin, DDT and its derivatives, imidacloprid, metalaxyl-M, atrazine, DCMU, and pyrimethanil, all of them found in high concentrations among Andean individuals. In these two groups of compounds, PNP (a metabolite of both ethyl parathion and methyl parathion) and methomyl (a metabolite of thiodicarb) are considered priority toxic contaminants by WHO (Classes Ia and Ib, respectively). This represents a cause for concern since these derivative metabolites were detected both with a high prevalence and concentration in Andean subjects. Furthermore, CPS, DDT, difenoconazole, imidacloprid, fipronil, metalaxyl-M, and tebuconazole (all Class II), which are included in the list of contaminants of particular concern to Peruvians, also pose a potential hazard to human health according to WHO.

While the WHO classification of pesticides by hazard is essentially based on the acute oral and dermal toxicity to rats, there is strong evidence that the priority toxic contaminants listed above present both acute and chronic risks to human and animal health. For example, methomyl is a widely used carbamate insecticide and one of the leading causes of accidental and suicidal poisoning in LMICs^37^. Through chronic sublethal exposure, methomyl is also a strong genotoxic agent that induces DNA damage and cytotoxicity^38^. Likewise, PNP is an endocrine disruptor that has oncogenic potential^39^. PNP has been reported to exert hepatotoxic effects in rodents by increasing liver transcription levels of genes encoding the estrogen receptor-α, glutathione S-transferase, and aryl hydrocarbon receptor signaling pathway^40^. An intriguing finding is that similar alterations in gene expression have been described in a molecular subtype of liver cancer developed by Peruvian patients originating from the Central Andes^20,41^, which might suggest a role for PNP in promoting the disease in the region.

Due to their hazards, certain pesticides have been banned over time in Peru. It is notably the case for DDT since 1991, pentachlorophenol since 1999, γ-HCH, ethyl parathion and methyl parathion since 2000, and methamidophos since 2020, according to the Peruvian National Food Safety and Quality Service (SENASA). Disturbingly, all of these ingredients or their derivative metabolites were detected in the present study. The detection of these compounds could be attributed to either illegal use or a low biodegradability, since their use had been prohibited, e.g., methamidophos, which was banned only a few months prior to our study. However, certain products have been banned for several decades and reports indicate that prohibited pesticides can still be purchased in Peru^18^, thus supporting the hypothesis of pesticide misuse. As an illustration of this situation, an outbreak involving parathion in Páucar del Sara Sara poisoned 111 people and killed nine of them in 2018, 19 years after the tragedy of Tauccamarca and 18 years after the prohibition of ethyl parathion and methyl parathion in Peru^17,18^. This alarming situation is thus corroborated herein by analyzing hair samples that uncover widespread and consistent exposure to pesticides in Andean subjects, although some of which have been deemed hazardous and banned by governmental authorities. Hence, our study highlights the need to enforce agrochemical use in Peru and develop guidelines for managing obsolete pesticide stocks.

In the present study, several additional issues are raised about how pesticide contamination originates in the Andean population. For instance, our multivariate analysis suggests that pesticide contamination of Peruvian subjects may not strictly be restricted to occupational or domestic activities such as farming and agrochemical use, as would be expected in the first place. Indeed, several authors have specifically addressed cases of intoxication among pesticide applicators in Peru^42,43^, however, our study shows alarming levels of pesticide contamination in non-users as well. Furthermore, the main source of exposure to pesticides does not appear to be contamination of clean water supplies for potable water, as previously evocated^44^. Without excluding this environmental factor, our multivariate analysis suggests that pesticide exposure occurs in the vicinity of where people live and the source of the food they consume. According to our analysis, a major risk of pesticide exposure for people living in rural areas would be eating food harvested directly from the field, most likely without having undergone effective decontamination such as washing with water or soaking in solutions of salt^45^. Although our geospatial modeling is constrained due to a lack of statistical power, it might suggest that the density of agrochemical use could be a proxy measure for assessing the risk of pesticide exposure to humans in Peru. Indeed, pesticide testing on human subjects on a regular basis, especially on a large scale, is intricate, since it entails ethical, methodological, and instrumental requirements^46^.

There are some limitations that are to be recognized in the present study. First, the number of subjects included might appear relatively small, particularly when considering the statistical power of our geospatial model. Here, we present the first explorative observational study on pesticide contamination in body samples from Andean people, and we had little knowledge about the magnitude of this contamination. That is why we decided to test a wide array of pesticide-related products, which experimentally resulted in limiting the number of subjects we included. Henceforth, this explorative study will provide a foundation for the detection of contaminants of interest in larger cohorts of subjects. Second, the types and levels of pesticide contamination may have been influenced by the dates of the study. To narrow this contingency, sample collection took place between November and December 2020, during the season in which pesticides are applied in the Andes from October to February. We took the time-lag dimension into account in our experimental design by testing hair samples long enough to enfold a three-month period^32,34^. The same strategy was applied to the French and Laotian subjects that we sampled in a time-lapse of six months surrounding the Peruvian sampling. Finally, because subjects self-reported the information retrospectively, there is a potential for recall bias. To restrain this bias, we administered a semistructured questionnaire that allowed information to be cross-checked^23,24^.

## Supplementary Information

Supplementary Information 1.

Supplementary Information 2.

## Data Availability

All data generated or analyzed during this study are included in this published article (and its supplementary information files).
