# Supplementary material for: Exposure assessment of 170 pesticide ingredients and derivative metabolites in people from the Central Andes of Peru
Source: Sci Rep. 2022 Aug 8;12:13525. doi: 10.1038/s41598-022-17772-1 (PMC9360020; doi:10.1038/s41598-022-17772-1)
Supplement: Supplementary file 1 — Supplementary Information 1. [file 41598_2022_17772_MOESM1_ESM.pdf]

The English version of the questionnaire follows the Spanish original version.

1 – DATOS DE LA ENCUESTA

1.1 Fecha en la que se completa el cuestionario (día/ mes/ año):

1.2 Hora de inicio de entrevista:

1.3 Lugar en el que se completa el cuestionario (Ciudad/ Provincia/ Departamento):

1.4 Coordenadas GPS (DMS):

2 – DATOS PERSONALES

Nombres :

Apellidos:

DNI:

Fecha de nacimiento (día/ mes/ año):

Edad: años Sexo: Hombre☐ Mujer☐

Lugar de nacimiento (Ciudad/ Provincia/ Departamento):

Teléfono:

Dirección actual:

3 – ENTORNO DE VIDA

3.1 Especificar los tipos de empresas o actividades principales o de importancia en su distrito (por ejemplos, minas, puertos, agrícolas, textiles, etc.):

**Tipos de actividades**

3.2 La comida que consume mayormente es: (una respuesta sola posible)
Marcar con una X lo que corresponda

Sembrada y cultivada	
Fresca del mercado	
Conserva o enlatada	
No conocido	

3.3 ¿Qué es lo que más consume? (puede marcar más de uno)
Marcar con una X los que correspondan

	Poco	Frecuentemente
Tubérculos (papas, camote, maca, etc.)		
Cereales		
Arroz		
Frutas		
Otro:		

3.4 ¿Si consume cereales, que tipo de cereales son? (puede marcar más de uno)
Marcar con una X los que correspondan

	Poco	Frecuentemente
Arroz		
Avena		
Cebada		
Kiwicha		
Kañiwa		
Maíz		
Quinua		
Trigo		
Otro:		

3.5 ¿De dónde viene el agua que consume mayormente? (una respuesta sola posible)
Marcar con una X lo que corresponda

Agua en botella	
Agua corriente/grifo	
Pozo	
Arroyo/rio	
Agua de lluvia	
Agua de cisterna	
No conocido	
Otro:	

3.6.1. ¿Utiliza usted o su familia insecticidas, fungicidas o herbicidas en casa?

Si	
No	

3.6.2. ¿Utiliza usted o su familia insecticidas, fungicidas o herbicidas en el trabajo?

Si	
No	

Si la respuesta es no en ambos casos, pasar a la pregunta 3.7.1.

3.6.3. Si usted o su familia utiliza insecticidas, fungicidas o herbicidas, ¿Con que frecuencia los utiliza? (una respuesta sola posible)
Marcar con una X lo que corresponda

Semanalmente	
Mensualmente	
Anualmente	
Otro (precisar):	

3.6.4 Si la respuesta es sí en las preguntas 3.6.1 y/o 3.6.2, por favor si posible nómbrelos:

**Nombres de las pesticidas**

3.7.1 ¿Tiene animales?

Si	
No	

Si la respuesta es no, pasar a las preguntas 3.8.1 y 3.8.2.

3.7.2 Si la respuesta a la pregunta es sí, ¿que tipo de animales tiene usted? (puede marcar más de uno)
Marcar con una X los que correspondan

Mascotas (perros, gatos, aves)	
Ganado (vacas, cerdos, ovejas, conejos)	
Aves de corral (pollos, patos, gansos)	
Cuyes	
Otro:	

3.7.3 Si tiene animales, por favor especificar que animales:

Tipos de animales

3.8.1. ¿Recibe tratamiento actualmente para alguna enfermedad?

Si	
No	

3.8.2 ¿Ha recibido tratamiento continuo para alguna enfermedad?

Si	
No	

Si la respuesta es no en ambos casos, pasar a la pregunta 3.9.1.

3.8.3 ¿Para cuales enfermedades ha recibo o recibe tratamientos continuos? Especifica los nombres de los tratamientos si puede.

Nombre de las enfermedades	Nombre de los tratamientos

3.9.1 ¿Utiliza usted champú para lavar sus cabellos?

Si	
No	

3.9.2 Si la respuesta a la pregunta es sí, ¿Con que frecuencia los utiliza champú? (una respuesta sola posible)
(Marcar con una X lo que corresponda)

Diario	
Cada 2 o 3 días	
Semanalmente	
Otro:	

3.9.3 ¿Ha utilizado tinte o decolorado sus cabellos en los últimos tres meses? (puede marcar más de uno)
(Marcar con una X lo que corresponda)

Ninguno	
Tinte	
Decoloración	

1.5 Hora de fin de entrevista:

1 – SURVEY DATA

1.1 Date on which the questionnaire is completed (day/ month/ year):

1.2 Interview start time:

1.3 Place where the questionnaire is completed (City/ Province/ Department):

1.4 GPS coordinates (DMS):

2 – PERSONAL INFORMATION

First names:

Surnames:

ID number:

Birthdate (day/ month/ year):

Age: years-old Gender: Man ☐ Woman ☐

Birthplace (City/ Province/ Department):

Phone number:

Current address:

3 – LIVING ENVIRONMENT

3.1 Specify the main types of activities or companies in your district (for examples, mines, ports, agriculture, textiles, etc.)

**Type of activities**

3.2 The food you eat the most is: (only one answer possible)
(Mark with an X what applies)

Planted and cultivated	
Fresh from the market	
Preserved or canned	
Not known	

3.3 What do you consume the most? (you can mark more than one)
(Mark with an X those that apply)

	Rarely	Frequently
Tubers (potatoes, sweet potatoes, Peruvian ginseng, etc.)		
Cereals		
Rice		
Fruits		
Other:		

3.4 If you eat cereals, what kind of cereals are they? (you can mark more than one)
(Mark with an X those that apply)

	Rarely	Frequently
Rice		
Oatmeal		
Barley		
Love-lies-bleeding		
Kaniwa		
Corn		
Quinoa		
Wheat		
Other:		

3.5 Where does the water you drink the most come from? (only one answer possible)
(Mark with an X what applies)

Bottled water	
Tapped water	
Well	
River	
Rain water	
Tanker water	
Not known	
Other:	

3.6.1. Do you or your family use insecticides, fungicides or herbicides at home?

Yes	
No	

3.6.2. Do you or your family use insecticides, fungicides or herbicides at work?

Yes	
No	

If the answer is no in both cases, go to question 3.7.1.

3.6.3. If you or your family use insecticides, fungicides or herbicides, how often do you use them? (only one answer possible)
(Mark with an X what applies)

Weekly	
Monthly	
Annually	
Other (specify):	

3.6.4 If the answer is yes in questions 3.6.1 and/or 3.6.2, please name them if possible:

**Pesticide names**

3.7.1 ¿Do you have animals?

Yes	
No	

If the answer is no, go to questions 3.8.1 and 3.8.2.

3.7.2 If the answer to the question is yes, what kind of animals do you have? (you can mark more than one)
(Mark with an X those that apply)

Pets (dogs, cats, birds)	
Livestock (cows, pigs, sheep, rabbits)	
Poultry (chicken, ducks, geese)	
Guinea pigs	
Other:	

3.7.3 If you have animals, please specify which animals:

**Types of animals**

3.8.1. Are you currently receiving treatment for any illness?

Yes	
No	

3.8.2 Have you received ongoing treatment for any illness?

Yes	
No	

If the answer is no in both cases, go to question 3.9.1.

3.8.3 For which illnesses have you received or do you receive continuous treatment? Specify the names of the treatments if you can.

**Name of the disease**	**Name of treatments**

3.9.1 Do you use shampoo to wash your hair?

Yes	
No	

3.9.2 If the answer to the question is yes, how often do you use shampoo? (only one answer possible)
(Mark with an X what applies)

Daily	
Every 2 or 3 days	
Weekly	
Other	

3.9.3 Have you dyed or bleached your hair in the last three months? (you can mark more than one)
(Mark with an X what applies)

None	
Dyeing	
Bleaching	

1.5 Interview end time:

Supplemental Figure 1

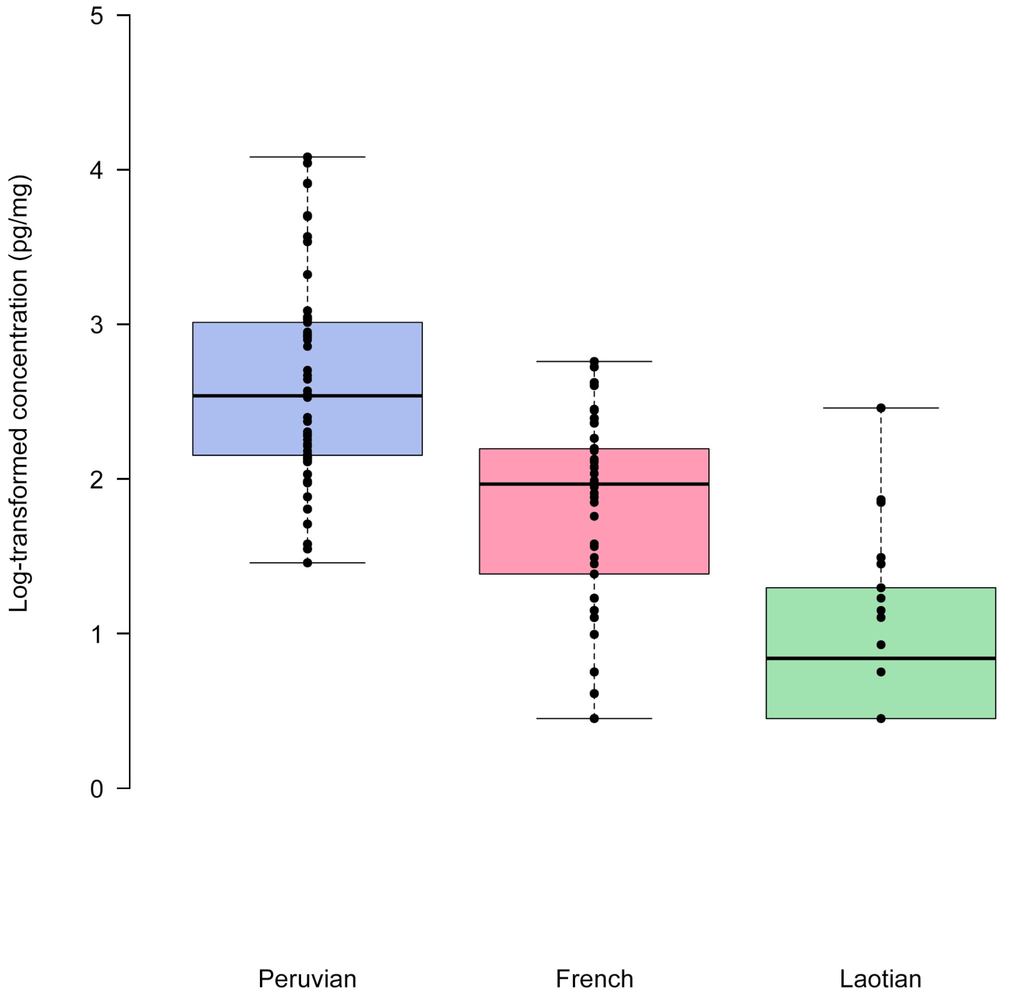

Box-and-whisker plot statistics

	Peruvian	French	Laotian
Upper whisker	2103.18	282.93	28.28
3rd quartile	1030.87	143.54	14.14
Median	345.99	81.07	2.83
1st quartile	142.56	11.31	2.83
Lower whisker	28.76	0.00	0.00
Number of data points	50	47	50

Box-and-whisker plots of the pesticide concentrations (pg/mg) measured in hair of French (*N* = 47; light red), Laotian (*N* = 50; light green), and Peruvian (*N* = 50; light blue) subjects. Upper part: Box plots are presented with log-transformed concentration data (for better visualization). Lower part: Table shows distribution statistics with raw concentration data (for better understanding).
